# Increase in use of psychiatric sick leave during COVID-19 pandemic by healthcare workers in a municipality in Argentina

**DOI:** 10.47626/1679-4435-2022-872

**Published:** 2022-03-30

**Authors:** Agustina María Marconi, Ursula Myers, Alfredo Mariano Retamar, Ivana Jazmin Freddi, Julieta Chiarelli, Rafael Zamora

**Affiliations:** 1 Epidemiology, University Health Services-University of Wisconsin, Madison, WI, USA.; 2 Psychiatry and Behavioral Sciences, Medical University of South Carolina, Charleston, SC, USA.; 3 CEMU Occupational Medicine, Vicente Lopez, Buenos Aires, Argentina.; 4 Latinas in Global Health, Miami, FL, USA.; 5 Epidemiology Area, Hospital Parmenio Piñero, Buenos Aires, Argentina.; 6 MEDICUS, Buenos Aires, Argentina.

**Keywords:** pandemics, health personnel, gender analysis, sick leave

## Abstract

**Introduction:**

The severe respiratory syndrome caused by the novel coronavirus has resulted in worldwide pressure on the healthcare workers attempting to treat millions of individuals ill with COVID-19, in addition to their regular duties.

**Objectives:**

To examine use of psychiatric leave by Argentinian healthcare workers during the COVID-19 pandemic, including exploring potential differences by sex.

**Methods:**

We analyzed psychiatric sick leave taken by municipal level healthcare workers in the Province of Buenos Aires, January - October 2020. We compared historical cases of psychiatric sick leave from 2015-2019 to leave requested in 2020.

**Results:**

Healthcare workers utilized 161.9% more psychiatric sick leave in 2020, with significantly more leave taken by women.

**Conclusions:**

Healthcare workers in the Argentinian municipality of Vicente Lopez took a significantly greater amount of psychiatric sick leave during the pandemic. The higher rates of psychiatric sick leave taken by women replicates other findings of higher rates of psychological symptoms in female healthcare workers.

## Introduction

The COVID-19 pandemic is the greatest global health crisis since the H1N1 influenza pandemic in the early twentieth century. In addition to direct health issues related to the virus, pandemics also lead to widespread anxiety, panic, and depression.^1,2^ Given the higher risk of exposure to the pathogen, shortages of equipment, extended workloads, and involvement in making emotional and ethical decisions, frontline and essential workers such as healthcare workers face increased stress, burnout, depression, and posttraumatic stress disorder.^3^

The severe respiratory syndrome caused by the novel coronavirus (severe acute respiratory syndrome coronavirus [SARS-CoV-2]) has caused worldwide pressure on the healthcare workers attempting to treat millions of individuals ill with COVID-19, in addition to their regular duties. As COVID-19 cases surged and everyone experienced stressors such as worries about pre-existing health conditions and childcare issues, healthcare workers also faced job-specific stressors including shortages of personal protective equipment, overwhelmed hospitals, lack of effective treatments, and having to decide who receives care and who does not.^4^ This can result in healthcare workers operating in ways that go against their personal and/or professional moral or ethical code, causing moral injury which in turn leads to increased psychological distress.^5^

Accordingly, a number of studies have found increased levels of anxiety and depression amongst international healthcare workers during the COVID-19 pandemic. A study conducted among medical staff at a tertiary infectious diseases hospital in China showed a high incidence of anxiety (23.04%) and stress disorders (27.39%).^6^ In Italy, the psychological pressure faced by healthcare frontline workers also resulted in increased rates of anxiety and depression.^7^ Compared to non-medical workers, Zhang et al.^8^ found higher prevalence of depression, anxiety, and insomnia among healthcare workers. A recent systematic review highlights some of the symptoms healthcare workers experienced during the pandemic months: 37.8% psychological distress (95% confidence interval [95%CI]: 28.4-48.2), 34.4% burnout (95%CI: 19.3-53.5), 29% anxiety features (95%CI: 14.2-50.3), 26.3% depressive symptoms (95%CI: 12.5-47.1), 20.7% posttraumatic stress disorder features (95%CI: 13.2-31%), 16.1% somatization (95%CI: 0.2-96.0%), and 14% stigmatization feelings (95%CI: 6.4-28.1).^9^

With regard to the impact of the pandemic on men versus women, despite a higher fatality rate from COVID-19 for men, women reported higher rates of mental health issues with an increased workload due to lockdown and quarantine measures.^10^ For example, young professionals, nurses, and women reported higher rates of psychological burden in Wuhan at the beginning of the pandemic.^11^ In fact, when examining burnout among healthcare workers, the only factor associated with high levels across domains was being female.^12^ Another recent review found that being younger, more junior, or being the primary caregiver of a young child increased the risk for psychological stress among healthcare workers during the pandemic.^13^

Outside of a pandemic, anxiety and depression are the most common mental health diagnoses among individuals in Argentina, the second largest country in Latin America, with 9.4% reporting anxiety and 5.7% reporting depression during an average 12-month period.^14^ Healthcare workers are a group of individuals who experience higher rates of mental health difficulties than the general population. Due to the nature of their work, healthcare workers report higher levels of anxiety, depression, and burnout and twice the rate of suicide compared to the general population.^15^ During the pandemic, 54% of Argentinean healthcare workers reported symptoms related to depression; importantly, 93% of the workers surveyed in this study reported they did not have any mental health diagnosis prior to the pandemic.^16^ What remains unknown is how these self-reported rates of anxiety and depression are impacting healthcare workers’ ability to continue to do their jobs. The aims of this project are to (1) examine use of psychiatric sick leave by Argentinian healthcare workers during the COVID-19 pandemic and (2) to explore if there were differences in psychiatric sick leave use by sex.

## Methods

### Setting

The Metropolitan Area of Buenos Aires is the common urban area that makes up the Autonomous City of Buenos Aires and 40 municipalities of the Province of Buenos Aires, including Vicente Lopez. The municipality of Vicente Lopez is located north of the Autonomous City of Buenos Aires and has a population of 270,929 inhabitants.^17^

### Data and analysis plan

All the data were provided by the Municipal Occupational Medicine Directorate. This Directorate routinely and systematically collects data on all absenteeism, sick leave, and personal vacations by municipal workers. For this analysis we focused on psychiatric sick leave for all healthcare workers. The research project was approved by the central ethics committee of the Province of Buenos Aires.

We conducted an exploratory analysis of “excess psychiatric sick leave” in healthcare workers at the Secretary of Health and Human Development for the Buenos Aires Province from January 2020 to October 2020. For the analysis we used cases of psychiatric sick leave for the same period over the previous 5 years (2015-2019) and observed psychiatric sick leave cases in 2020. We report the results in rates per 100 healthcare workers.

We performed the analysis for total psychiatric sick leave and stratified by sex. We analyzed the sick leave rate per the whole period as well as per month. We compared observed rates for the current period with the average expected psychiatric sick leave and the upper limit of the 95% confidence interval (95%CI) derived from the 5 years of historic data. An Institutional Review Board (IRB 2873) approved the protocol.

### Calculation of excess psychiatric sick leave

To measure excess psychiatric sick leave during the COVID-19 pandemic, we used: (1) Psychiatric sick leave expected to have occurred on a monthly basis based on the same period over the past 5 years (based on historical data), and (2) Psychiatric sick leave that actually occurred/was observed in the period analyzed. To assess the difference in “excess psychiatric sick leave” between women and men we determined the difference in proportions for both groups and compared the average expected proportion and the upper limit of the 95CI% derived from the five years of historic psychiatric sick leave data. We used the following operational definitions for the different categories of leave: (a) Sick leave: an absence from work permitted because of illness. All absences are paid. Regardless of the event itself, each healthcare worker has a maximum of 90 days per year if they have worked in the Municipality for less than 5 years. For workers with more than 5 years, the total amount of days per year increases to 180 days. When the worker is in charge of a family member (children, parents, partner) the allowance is doubled to 180 days per year and 360 days per year. Psychiatric sick leave is included in medical sick leave. (b) Psychiatric sick leave: A subset of sick leave that healthcare workers can designate to describe the leave that they are requesting. Most Occupational Medicine Directorates in Argentina label sick leave requests in categories, one of which is psychiatric sick leave.

## Results

**Figure 1:**
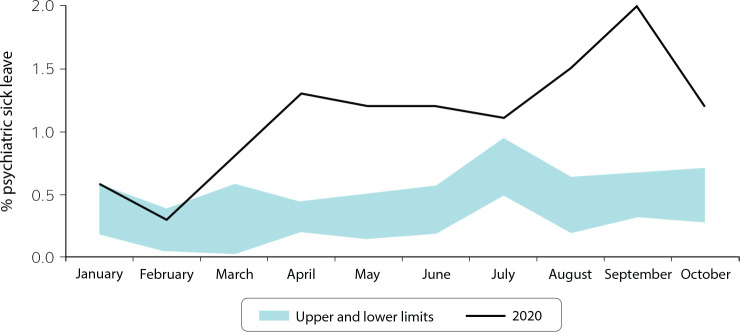
Psychiatric sick leave in healthcare workers, total sample per month. 2015-2019 upper and lower 95% confidence interval limits versus 2020. Vicente Lopez, Argentina. January-October.

The psychiatric sick leave rate per 100 healthcare workers for the whole period analyzed in 2020 was 1.10%, compared to the historical data from 2015-2019, which had an average psychiatric sick leave rate per 100 healthcare workers of 0.42% (Table 1 and Figure 1). In other words, healthcare workers took 161.9% more psychiatric sick leave in 2020, compared to previous years (95%CI: 122.97%). When examining the data per month, psychiatric sick leave use was higher than the historical data from February 2020 onwards (when the first reports of COVID-19 were being published widely). Overall, the month with the highest increase in psychiatric sick leave compared to the historical data was April 2020, when healthcare workers took 306.25% more psychiatric sick leave.

**Table 1:** Historical comparison of excess psychiatric sick leave in healthcare workers. Total and per month. Vicente Lopez, Argentina. January-October 2020

Month	2015-2019 monthly average × 100 healthcare workers (95%CI)	2020 psychiatric sick leave × 100 healthcare workers	% increase above baseline	% increase above threshold
January	0.38 (0.17-0.59)	0.60	57.89	1.82
February	0.22 (0.05-0.39)	0.30	36.36	-22.80
March	0.30 (0.02-0.58)	0.80	166.67	38.60
April	0.32 (0.21-0.43)	1.30	306.25	199.34
May	0.32 (0.15-0.49)	1.20	275.00	145.60
June	0.38 (0.20-0.56)	1.20	215.79	114.42
July	0.72(0.48-0.96)	1.10	52.78	15.16
August	0.42 (0.19-0.65)	1.50	257.14	131.88
September	0.50 (0.32-0.68)	2.00	300.00	196.16
October	0.50 (0.29-0.71)	1.20	140.00	70.08
Total	0.42 (0.35-0.49)	1.10	161.20	122.97

95%CI = 95% confidence interval.

**Figure 2:**
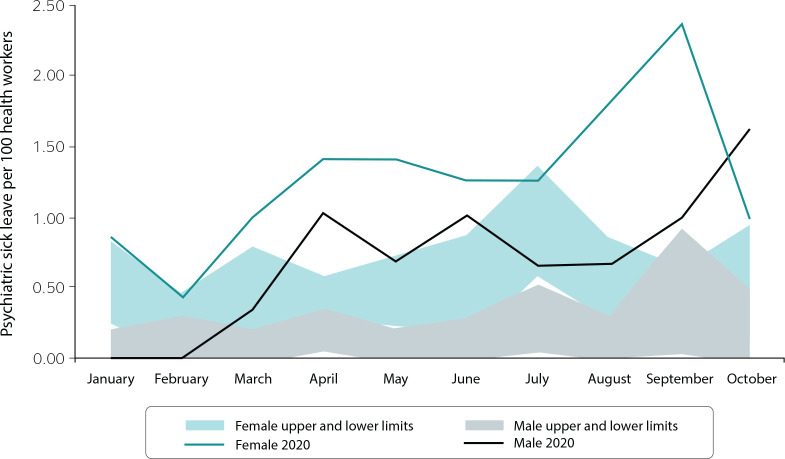
Psychiatric sick leave in healthcare workers, by sex and per month. 2015-2019 upper and lower 95% confidence interval limits versus 2020. Vicente Lopez, Argentina. January-October.

When stratifying the monthly psychiatric sick leave rate by sex (Table 2 and Figure 2), female healthcare workers had a 1.3% rate in 2020 compared to the 0.53% per 100 in 2015-2019, an increase of 137.8% more psychiatric leave (95%CI: 98.8%). Comparatively, male healthcare workers had a psychiatric sick leave rate of 0.70% in 2020, a substantial increase from the 0.17% rate in the historical data (a 307% increase, 95%CI: 152.9%). Monthly, there were differences in which group utilized more psychiatric sick leave. While women took significantly more psychiatric sick in all months except for February and July, the largest increase in use was in September 2020, which was 345.3% higher than the historical data. Men, on the other hand, had significant increases in psychiatric sick leave in all months after February 2020, with the largest increase in May 2020, when the rate was almost 910% higher than the historical data.

**Table 2:** Historical comparison of excess psychiatric sick leave in healthcare workers. Total and per month by sex. Vicente Lopez, Argentina. January-October 2020

Sex	Month	2015-2019 monthly average × 100 healthcare workers (95%CI)	2020 psychiatric sick leave × 100 healthcare workers	% increase above baseline	% increase above threshold
Female	January	0.54 (0.24-0.84)	0.86	59.85	2.87
	February	0.26(0.06-0.46)	0.43	65.38	-5.84
	March	0.43 (0.07-0.79)	0.99	132.39	26.07
	April	0.41 (0.25-0.58)	1.41	240.58	143.23
	May	0.45 (0.21-0.70)	1.4	209.73	101.20
	June	0.53 (0.20-0.87)	1.26	136.84	45.64
	July	0.96 (0.57-1.36)	1.25	29.94	-7.97
	August	0.57 (0.28-0.86)	1.81	218.66	110.31
	September	0.53 (0.43-0.63)	2.36	345.28	276.15
	October	0.68 (0.43-0.64)	0.97	43.07	3.42
Total female		0.53 (0.43-0.64)	1.27	137.83	98.77
Male	January	0.07 (-0.06-0.19)	0.00	-100.00	-100.00
	February	0.13 (-0.03-0.30)	0.00	-100.00	-100.00
	March	0.07 (-0.07-0.20)	0.34	400.00	68.92
	April	0.20 (0.04-0.35)	1.02	418.43	187.88
	May	0.07 (-0.06-0.20)	0.68	909.80	241.15
	June	0.13(-0.03-0.30)	1.01	676.94	253.09
	July	0.27 (0.02-0.51)	0.66	146.09	28.28
	August	0.14 (-0.03-0.30)	0.66	383.65	119.82
	September	0.46 (0.02-0.91)	0.98	112.18	8.25
	October	0.20 (-0.06-0.47)	1.63	706.03	248.18
Total male		0.17 (0.07-0.28)	0.70	306.98	152.97

95%CI = 95% confidence interval.

**Figure 3:**
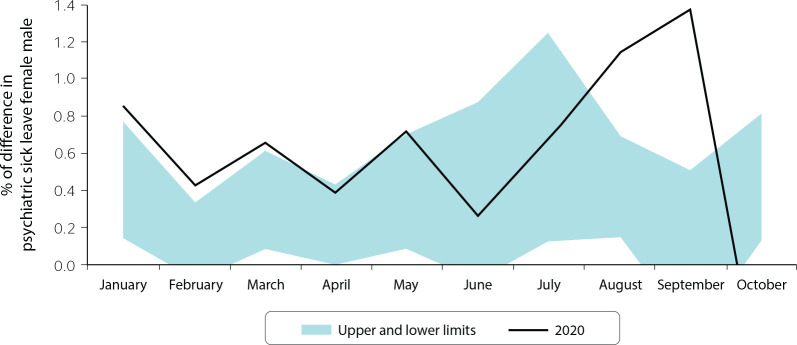
Difference in percentage psychiatric sick leave by sex in healthcare workers: female-male. 2015-2019 upper and lower 95% confidence interval limits versus 2020. Vicente Lopez, Argentina. January-October.

We found significant differences in the proportion of psychiatric sick leave taken by women versus men during the pandemic (Table 3 and Figure 3). Women utilized 59.34% more psychiatric sick leave than their male counterparts (95%CI: 22.93%). This result was mainly driven by greater amounts of leave taken by women in January, February, March, May, August, and September (which had the largest increase in use by women). Men only took more psychiatric sick leave than women in October 2020.

**Table 3:** Historical comparison of difference in proportion of psychiatric sick leave by sex in healthcare workers. Total and per month. Vicente Lopez, Argentina. January-October 2020

Month	2015-2019 difference in proportion Monthly average × 100 (95%CI)	2020 difference in proportion × 100	% increase above baseline	% increase above threshold
January	0.47 (0.16-0.78)	0.86	82.20	10.19
February	0.13 (-0.08-0.33)	0.43	241.27	28.73
March	0.36 (0.10-0.62)	0.66	84.36	6.67
April	0.22 (0.00-0.43)	0.39	80.56	-9.89
May	0.39 (0.08-0.69)	0.72	86.53	3.91
June	0.40 (-0.08-0.88)	0.25	-37.81	-71.62
July	0.69 (0.13-1.25)	0.59	-14.99	-52.92
August	0.43 (0.17-0.70)	1.15	164.98	63.90
September	0.07 (-0.38-0.51)	1.38	1990.91	169.02
October	0.48(-0.14-0.81)	-0.66	-238.66	-181.29
Total	0.36 (0.26-0.47)	0.58	59.34	22.93

95%CI = 95% confidence interval.

## Discussion

In line with recent findings of increased anxiety and depression in healthcare workers,^18,19^ this study found healthcare workers in the Argentinian municipality of Vicente Lopez took significantly more psychiatric sick leave during the first eight months of the COVID-19 pandemic compared to previous years, with the largest increase occurring in April 2020, when uncertainty surrounding COVID-19 was at its highest globally. Similar to other outbreaks such as SARS in 2003, the uncertainty surrounding COVID-19 including lack of information about transmission, treatment, and mortality rates likely led to healthcare workers feeling higher rates of stress, leading to them take psychiatric sick leave.^20^

A number of different factors impacted healthcare providers’ use of psychiatric leave differentially, such as the higher rates of psychiatric sick leave used by female healthcare workers compared to their male counterparts. This replicates findings of personal and work-related burnout^21^ as well as higher rates of reported psychological symptoms^22^ in female healthcare workers. It is possible that, due to traditional gender roles, these female healthcare workers may have been primarily responsible for childcare as well as for caring for elderly people, which may have increased their levels of stress and resulted in more psychiatric sick leave being taken. Parenting responsibilities have been reported as stressors during the current COVID-19 pandemic^23,24^ as well as in the SARS outbreak in 2003.^25^ Alternatively, it is also possible that the impact of masculinity on healthcare avoidance may have resulted in men taking a similar amount of leave, but labeling it differently compared to their female counterparts.^26^

Increased used of either general or psychiatric sick leave reflects challenges experienced by healthcare workers individually and the healthcare system as a whole. On an individual level, while removing the underlying stressor of the pandemic is impossible, there are interventions for anxiety and depression, such as Psychological First Aid and Skills for Psychological Recovery, that could result in fewer days off in the long-term as healthcare workers develop skills to help cope with the increased stress. For healthcare teams, increases in sick leave mean staff shortages during peak-capacity periods, resulting in increased demand and unplanned shifts for the remaining workers.^27^

This increased burden can then increase the risk of those individuals needing their own leave in the future, continuing to leave the team short-staffed. Without intervention, systems can begin to buckle under the weight of an overburdened healthcare workforce. There are a number of potential interventions healthcare systems could implement to aid healthcare workers. Telehealth, for example, has increased during the pandemic to approximately 1 billion appointments a year, compared to the originally projected 36 million appointments prior to COVID-19.^28^

Strategic use of telehealth appointments, both for outpatient appointments that can be conducted via telehealth fairly easily (e.g., mental health appointments) and for triage of care via telehealth from overstretched hospitals in a virus hotspot to other facilities, would allow for more even workflow and may prevent healthcare workers from feeling burnout as rapidly. Additionally, allowing providers to telework from home when possible could result in lower levels of anxiety related to virus exposure.

This study has a number of limitations that are important to consider when interpreting the results. First, many countries do not have different categories of leave, preventing comparison of our results with samples internationally. Even with the categorizing of psychiatric versus general leave in our sample, we do not know what individual episodes or diagnoses were associated with taking leave. Within this sample, we also do not know which hospitals or units these healthcare workers were assigned to or their levels of exposure to patients with COVID-19, preventing us from looking at the direction of the relationship between caring for individuals with COVID-19 and use of psychiatric leave by healthcare workers. Finally, data on the age of the healthcare workers were not available for this project. As other projects have found that younger healthcare workers have reported higher rates of mental health problems during the pandemic,^21^ this would have been interesting to replicate in our sample.

Despite these limitations, this study adds important information about how healthcare workers have been responding to the COVID-19 pandemic. As expected, healthcare workers utilized higher rates of psychiatric leave while struggling with increased stress as a result of personal, institutional, and global factors. For healthcare workers who experience stress, individual interventions such as Psychological First Aid could provide coping skills. However, beyond the individual level, there are a number of systemic changes a healthcare system could make, including using telehealth as a way of reducing the burden on overstretched hospitals and for anxious healthcare providers, resulting in fewer days of psychiatric leave being taken.

### Future proposals

The data shows a flip in October in the difference within sexes, with male healthcare workers’ leave exceeding leave among female workers. It would be interesting to see if this pattern continues in the following months up to the end of 2020 and at the start of 2021.

### Consent for publication

No identifiable data was utilized in this analysis.
